# Dietary supplementation of capsaicin on production performance, egg quality, follicle development and liver metabolism of late-phase Changshun green-shell laying hens

**DOI:** 10.1016/j.psj.2025.105841

**Published:** 2025-09-11

**Authors:** Bolin Zhang, Ning Liu, Xueping Shi, Caichao Zhang, Yingchun Liu, Di Yang, Zongzheng Liu, Wei Wang, Shubai Wang

**Affiliations:** a College of Animal Science and Technology, Qingdao Agricultural University, No. 700, Chang Cheng Road, Cheng Yang District, Qingdao 266109, People's Republic of China; b Department of Biology and Agriculture, Zunyi Normal College, Ping`an Avenue, Hong Huagang District, Zunyi 563006, People's Republic of China; c College of Animal Science and Technology, Jilin Agricultural University, No. 2888, Xincheng Road, Jingyue District, Changchun 130118, People's Republic of China; d Animal Health and Product Quality Supervision Station of Pingdu, No.1 Zhenxing Road, Guozhuang, Nancun Town, Pingdu 266736, People's Republic of China; e Qingdao Livestock Workstation, No.149, Xia Zhuang Road, Li Cang District, Qingdao 266071, People's Republic of China

**Keywords:** Capsaicin, Reproduction performance, Gut microbial, Follicle development, Liver lipid metabolism, Laying hens

## Abstract

This study aimed to investigate the impacts of dietary capsaicin (**CAP**) on production performance, egg quality, follicle development, anti-oxidant capacity and lipid metabolism of laying hens at the late-period laying cycle. A total of 240 healthy Changshun green-shell late-phase laying hens were chosen and randomly allocated to three treatments, each consisted of 10 replicates with 8 hens per replicate. The hens in the control group were freely access to the basal diet and hens in the CAP group freely received diets containing 150 mg/kg CAP. While hens in the pair-feed (**PF**) group receiving the basal diet with amounts equal to those consumed the CAP diet on the previous day. The results showed that CAP supplementation increased egg mass and egg production of laying hens as well as improved egg yolk color and Haugh unit (*P* < 0.05). Furthermore, CAP supplementation reduced the concentrations of total cholesterol (**TC**), triglyceride (**TG**) and low-density lipoprotein cholesterol (**LDL-C**) in plasma (*P* < 0.05). But CAP supplementation elevated the concentrations of follicle stimulating hormone releasing hormone (**FSH**), luteinizing hormone (**LH**), progesterone (**P4**) and Estradiol (**E2**) in serum (*P* < 0.05). CAP addition downregulated mRNA expressions of acetyl-CoA carboxylase (**ACC**), fatty-acid synthase (**FAS**), sterol regulatory element binding protein-1 (**SREBP1**) and liver X receptors (**LXRs**, *P* < 0.05) in liver. In addition, CAP addition increased the abundances of *Bacillota, Actinomycetota, and Puseudomonadota* in the cecum of laying hens (*P* < 0.05). Besides, the concentrations of propionic acid, butyric acid, isobutyric acid and caproic acid were higher in the CAP group (*P* < 0.05). Additionally, CAP also upregulated mRNA expressions of transient receptor potential cation channel subfamily V member 1 (***TRPV1***), calmodulin (***CaM***), follicle stimulating hormone receptor (***FSHR***) and luteinizing hormone receptor (***LHR***) in ovary (*P* < 0.05) and downregulated related mRNA expressions involved in lipid metabolism in the liver of layers (*P* < 0.05). Collectively, these results demonstrated that CAP supplementation could increase the production performance, improve egg quality, promote follicle development of late-phase laying hens by altering cecal microbiota and metabolism, liver lipid metabolism and oviduct function.

## Introduction

The layer industry is one of the key components contributing sustainable animal-derived food sources and the economic worth of its output is determined by both the quantity and quality of eggs (Chen et al., 2024). However, during the late stage of laying period, the physiological functions of reproductive system of hens decline accompanied by the decreases in secreted reproductive hormones, less follicle number associated with the ovarian aging and hypofunction (Chen et al., 2021a; Hao et al., 2021). Moreover, it has been demonstrated that a sharp drop in egg production and poor egg quality is observed in laying hens at the end of the laying cycle, which is closely associated with the suppression of follicular development and ovarian oxidative stress (Attia et al., 2020). Therefore, nutritional intervention is required to improve late-stage egg production and quality traits in laying hens.

Chili pepper is one of the most economically important genus, which has been used as food vegetables, natural colorants and in traditional medicines (Hernández‐Pérez et al., 2020). It is well known that chili pepper contains outstanding bioactive compounds such as flavonoids, carotenoids and capsaicinoids, the major active compound proved to owing a positive role in health (Hernández‐Pérez et al., 2020). In a previous study, it was found that 1 % red pepper powder supplemented in diet promoted the development of reproductive organs and follicles compared with the control group (Özer et al., 2005). About-Elkhari et al. (2018) demonstrated that dietary 0.5 % red pepper powder supplementation significantly increased egg production rate of laying hens, suggesting that red pepper powder could contribute to improving production performance of laying hens. Capsaicin (**CAP**) is the active ingredient in chili peppers of the family Capsicum (Guler and Zik, 2020). Accumulated evidences suggested that CAP derived from chili pepper has a variety of biological activities, including enhancing the antioxidant capacity, downregulating inflammatory cytokines and regulating lipid metabolism (Abou-Elkhair et al., 2018; Abd El-Hack et al., 2022). Additionally, a previous study demonstrated that 150 mg/kg CAP supplementation in diet tended to increase egg production and egg weight in 58-week-old laying ducks. Moreover, the relative weights of follicle development including large yellow follicles (**LYF**) and small yellow follicles (**SYF**) were significantly higher in the CAP-supplemented group than those in the control group (Liu et al., 2021a), suggesting that CAP supplementation may promote follicular development. and modulating the gut microbiota. Considering the numerousbenefits of CAP to the poultry industry, it may serve as a targeted plant-derived compound to improve production performance, promote follicle development and regulate lipid metabolism of hens in the late phase laying cycle. Moreover, our previous study demonstrated that aging layers in terminal production receiving diets supplemented with 150 mg/kg CAP exhibited an increased feed intake and a lower feed to egg ratio, the improved production performance and egg quality combined with the enhanced reproductive hormones concentrations in serum (Zhang et al., 2024). However, little information is known about the regulation of CAP supplementation on anti-oxidant capacity, lipid metabolism and gut microbiota of layers during the late laying period. Based on the information mentioned above, we hypothesized that dietary CAP supplementation could modulate gut intestinal microbiota, improve lipid metabolism, ameliorate ovary health and regulate oviduct-specific gene expressions, thereby enhancing production performance and egg quality in aging hens.

Therefore, the objective of the current study was to investigate the effects of CAP supplementation on production performance, egg quality, antioxidant capacity, intestinal microbiota and liver lipid metabolism, subsequently revealing the regulation mechanism of CAP on follicle development of Changshun green-shell laying hens in late-phase laying period.

## Materials and methods

### Animals and experimental design

All experimental protocols were approved by the Institutional Animal Care and Use Committee of Qingdao agricultural university (QAU1121010) and strictly followed the committee`s ethical standards.

A total of 240 healthy Changshun green-shell laying hens (60 weeks of age) with the similar laying performance and body weight were chosen and randomly allocated to three treatments, each containing 10 replicates and 8 hens per replicate. The hens in the control group were freely access to the corn-soybean basal diet and hens in the CAP group freely received diets containing 150 mg/kg CAP purchased from Fangben Biotechnology Co. Ltd. (Zunyi, Guizhou, China). While hens in the pair-feed (**PF**) group received the basal diet with amounts equal to those consumed by the CAP group on the previous day. The diets were formulated nutritionally balanced to fully satisfy the nutrient specifications recommended in the Chinese Feeding Standard of Chicken. The composition and nutrient levels in the basal diet were shown in Table 1. The light regimen was 16 h light and 8 h dark. Hens were freely access to water in the whole experiment. The experimental duration was 56 days.

**Table 1:** Composition and nutrient levels of the basal diet (air-dry basis, %).

Items	Contents	Nutrient level	Contents
Corn	56.50	Crude protein (%)	16.72
Soybean meal, 45.8 % CP	13.30	Metabolizable energy (MJ/kg)	11.38
Corn gluten meal, 63.5 % CP	3.85	Lysine (%)	0.75
Wheat bran, 12.5 % CP	3.77	Methionine + Cystine (%)	0.65
Soybean oil	2.30	Calcium (%)	3.50
Rapeseed meal, 35 % CP	8.85	Available phosphorus (%)	0.33
L-Lysine HCl	0.06		
DL-Methionine	0.05		
Calcium monophosphate	1.30		
Limestone	8.60		
Choline chloride	0.10		
Phytase	0.02		
Salt	0.30		
1Premix	1.00		

1 Premix provided the following per kg of the diet::vitamin A 11,800 IU;vitamin D_3_ 3,500 IU;vitamin E 5.00 mg;vitamin K_3_ 1.40 mg;vitamin B_1_ 2.80 mg;vitamin B_2_ 5.5 mg;nicotinamide 20 mg;D-calcium pantothenate 2.20 mg;vitamin B_6_ 3.00 mg;biotin 0.10 mg;folic acid 0.25 mg;vitamin B_12_ 5.0 mg;Fe (as ferrous sulfate) 65.0 mg;Cu (as copper sulfate) 10.0 mg;Mn (as manganese sulfate) 66 mg;Zn (as zinc sulfate) 80 mg;I (as potassium iodide) 0.35 mg;Se (as sodium selenite) 0.30 mg. ^2^Metabolizable energy and available phosphorus were calculated values based on diet composition, while the others were measured values. ^3^CP, crude protein.

### Sample collection

Following an 8-hour feed deprivation on the day before the end of the experiment, 10 hens from each treatment were randomly selected to collect blood from wing vein. The blood samples were collected into tubes coated with and without EDTA, followed by a centrifugation at 4°C 3000 × *g* for 10 min to collect plasma and serum for further analysis. After blood sample collection, hens were executed with humanitarian slaughter and the cecal contents were collected into sterile tubes which were stored in liquid nitrogen for metagenome analysis of gut microbiota. In addition, tissue samples from the ovary and liver of each hen in the three treatments were separately collected into frozen-tubes stored in liquid nitrogen for further analysis.

### Production performance

The numbers of egg production, total egg weight and feed intake were daily recorded to calculate the percentage of egg production, the average egg weight, laying percentage, average daily feed intake (**ADFI**) and the ratio of feed to egg weight (**FCR**). Egg mass was calculated by multiplying each egg weight by the percentage of egg production.

### Determination of egg quality

At the last day of each two week during the entire experiment, 20 eggs from each treatment were collected for egg quality analysis in 48 h after production. The parameters for egg shape index, yolk ratio and album ratio were determined according to Liu et al. (2021b). The album height was evaluated with Digital analyzer of album height (EQ-1A, Beijing Tianxiang Tianyu Technology Co., Ltd., Beijing, China) and yolk color was analyzed with 15-grade DSM Roche yolk color fan.

### Biochemical parameters in blood samples

Lipid profiles including total cholesterol (**TC**), triglycerides (**TG**), high density lipoprotein cholesterol (**HDL-C**), low density lipoprotein cholesterol (**LDL-C**) in plasma were determined. Antioxidant capacities were assessed total antioxidant capacity (**T-AOC**), superoxide dismutase (**SOD**), glutathione peroxidase (**GSH-Px**) and malondialdehyde (**MDA**) in plasma. The contents reproductive hormones including follicle stimulating hormone (**FSH**), estradio-17β **(E2**), luteinizing hormone (**LH**) and progesterone (**P4**) in serum. These blood parameters were determined with commercial kits (Nanjing Jiancheng Bioengineering Institute, Nanjing, China) according to manufacturer`s instructions.

### Transcriptome sequencing

Total RNA extracted from liver was conducted using TRIZOL reagent, followed by RNA purity and integrity analysis using 1 % agarose gel electrophoresis and Bioanalyzer 2100 system (Agilent Technologies,Co., Ltd., Palo Alto, CA), respectively. Sequencing libraries were constructed using NEBNext Ultra Directional RNA Library Prep Kit for Illumina (New England Bioscience, Ipswich, MA) (Chu et al., 2025). The quality control of the clean reads was done using the Trimmomatic v0.33 with the default parameters and then located to the reference genome of chickens using Hisat2 software (https://asia.ensembl.org/Gallus_gallus/Info/Index?db=core). Statistical analysis was performed by DESeq2 based on negative binomial distribution, and differential mRNA screen was conducted according to the threshold *P* < 0.05 and log2(FC)>1. Gene Ontology (**GO**) and Kyoto Encyclopedia of Genes and Genomes (**KEGG**) enrichment analyses were performed using the R package cluster Profiler v4.0 and ggplot2 (Wu et al., 2021).

### Cecal fatty acids analysis using UPLC-MS/MS

500 μL cold extraction solvent (methanol: acetonitrile: water =2:2:1, v/v/v) was added to equivalent frozen and the samples were homogenized at 4°C. After incubation on ice for an additional 20 min, the tissue extract was centrifuged for 10 min at 14,000 g and 4°C. Supernatants were collected into tubes and thoroughly freeze-dried. The samples were reconstituted in 50 μL of methanol water (1:1, v/v) before UPLC-MS/MS measurement. Isotopologue distribution was analyzed by a Thermo Q-Exactive Plus quadrupole-Orbitrap MS coupled to a Thermo Vanquish UPLC system (Thermo Scientific, San Jose, CA). The quantification of metabolites was conducted via triple quadrupole mass spectrometry operated in multiple reaction monitoring (MRM) mode, with subsequent identification carried out using the Met Ware database alongside public repositories such as HMDB, KEGG, and METLIN, by matching accurate m/z values, retention times, and fragmentation spectra. Mass spectrometry data processing was performed using Analyst software (v1.6.1, AB SCIEX, ON, Canada). After acquiring mass spectra, the peak data were processed, aligned, and normalized across all samples. The processed metabolite data were then subjected to both principal component analysis (**PCA**) and orthogonal partial least squares-discriminant analysis (**OPLS-DDA**) to visualize global patterns and identify group-specific metabolic markers, respectively. Differentially expressed metabolites (**DEMs**) were screened by combining VIP scores from the OPLS-DA model with univariate fold-change thresholds (≥2 or ≤0.5), using a VIP ≥ 1 as an additional cutoff. Significant DEMs were further evaluated through hierarchical clustering and correlation analysis. Subsequently, the underlying metabolic pathways were then elucidated using the KEGG and MetaboAnalyst databases.

### Microbial DNA extraction and metagenomics analysis

Cecal samples were collected, immediately frozen at −80°C, and later analyzed for microbial abundance via metagenomic sequencing. Total microbial genomic DNA was extracted from cecal samples using the E.Z.N.A™ Mag-Bind Stool DNA Kit (Omega Bio-tek Inc., Norcross, GA). The amplification products were sequenced on the Illumina MiSeq platform (Illumina, San Diego, CA) to construct DNA libraries according to the standard protocol. DNA libraries were prepared using the NEBNext Ultra DNA Library Prep Kit for Illumina (New England Biolabs Inc., Ipswich, MA), with quality assessed on an Agilent 2100 Bioanalyzer (Agilent Technologies, Santa Clara, CA). Whole-genome sequencing was performed on an Illumina NovaSeq 6000 system (Illumina Inc., San Diego, CA).

The raw sequencing data underwent quality control processing to yield valid reads for subsequent analysis. Firstly, sequencing adapters were trimmed from raw reads employing Cutadapt v1.9 (Kechin et al., 2017). Secondly, a sliding-window algorithm in FQTRIM (v0.94) was applied to remove low-quality reads. Subsequently, quality control was performed using FASTP to eliminate low-quality reads and contaminated reads, including sequences related to host and laboratory filtered by the alignment tool Bowtie2. Kraken2 was employed for metagenomic classification based on reference databases, consisting of 332,371 bacterial, 1934 archaeal and 18,639 viral reference genomes from the NCBI Ref Seq database and 3,047 fungal reference genomes from the NCBI Ref Seq database (Wood et al., 2019). Then α- and β diversity analysis were performed, and the statistical comparison of differences in α-diversity between the groups were conducted via the Wilcoxon rank test in the R package “Ggpubr” (v0.4.0). Species composition bar charts were analyzed using R (v4.4.1) based on species annotation and relative abundance results. Microbial community differences between groups were assessed using linear discriminant analysis effect size (**LEfSe**), with only taxa demonstrating linear discriminant analysis (**LDA**) scores >3.0 considered significant. Only bacterial taxa with LDA scores exceeding the threshold of 3.0 were displayed. Gut microbiota-metabolic indicator correlations were determined by a two-tailed Spearman's rank test.

### RNA isolation and RT-PCR analysis

The protocol of RNA extraction and RT-PCR analysis were conducted according to our previous study (Liu et al., 2025). RNA extraction from frozen liver and follicles were separately isolated using Trizol (Invitrogen Life Technologies, Carlsbad, CA), followed by the identification of RNA contents and purity, which was subsequently reversed into cDNA. The real-time PCR program consisted of one cycle at 95°C for 30 s, 40 cycles at 95°C for 5 s and 60°C for 30 s (Zhang et al., 2022). The sequence-specific primers were presented in Table 2. RT-PCR analysis for mRNA expressions of *ACC, FAS, SREBP1, CYP7A1, PPARα* and *LXRs* were conducted in samples from the liver. While RT-PCR analysis for mRNA expressions of *TRPV1, CaM, StAR, CYP19A1, CYP11A1, FSHR, LHR, Bax* and *Bcl2* were conducted in samples from follicles. Technical triplicates were run for all samples, and relative gene expression levels (normalized to β-actin as the reference gene) were determined using 2^-△△Ct^ method (Livak and Schmittgen, 2001).

**Table 2:** Sequence-specific primers for real-time quantitative PCR analysis.

Genes	Primers (5′→3′)	Amplicon size	Genebank accession
*FAS*	Forward: AACCCTACTAAGCAGCCTGAGAATG Reverse: TTGTCCGCCTTCCTGATTGCTAG	103 bp	XM_027459847.2
*ACC*	Forward: GGAGGAGGAGGGAAGGGAAT Reverse: GGAGCAATCCCGACCAAAGA	195 bp	XM_015295697.4
*SREBP1*	Forward: GAGACCATCTACAGCTCCGC Reverse: CATCCGAAAAGCACCCCTCT	154 bp	NM_204126.3
*CYP7A1*	Forward: GCTCTGCTCCACAAGAACCT Reverse: CCACGTGGGTCTTTGCTTTT	121 bp	NM_001001753.2
*CaM*	Forward: TGAAGTAGACGCTGATGGCAA Reverse: ACACACGGAACGCTTCTCT	116 bp	NM_205005.2
*LXRs*	Forward: TCACTCTGAACAGGGGTGGA Reverse: CACTCACCTTGGAACACATGC	132 bp	XM_015286920.2
*FSHR*	Forward: GTGACACCAAGATTGCCAAGCG Reverse: ACACTGTGATGAGAGGAACCTTGAG	117 bp	XM_021267215.1
*LHR*	Forward: TTCTCTGCTTACACCCCCAACTG Reverse: TTGCACAGCTTCTCTGAAGCTGAC	104 bp	XM_021267245.1
*TRPV1*	Forward: GCAGCCATCCAAAAGCAACA Reverse: TCCCCCTCTCCATATTGGCA	152 bp	NM_204572.1
*PPARα*	Forward: ATGCCAAGGTCTGAGAAGGC Reverse: CCCTGCAAGGATGACTCTGG	168 bp	NM_001001464.1
*StAR*	Forward: GGTGGACAACATGGAGCAGA Reverse: CAGAGTGTCCTTCCCAACCC	85 bp	NM_204686.3
*CYP11A1*	Forward: TGAATATCATCAGCCCCCGC Reverse: GTAGGGCTTGTTGCGGTAGT	116 bp	NM_001001756.2
*CYP19A1*	Forward: CGGGGCTGTGTAGGAAAGTT Reverse: AAACCATCTCCAGCAGAGGC	172 bp	NM_001001761.4
*Bax*	Forward: TCCTCATCGCCATGCTCATC Reverse: CATGTGGAGCCAAGGGACAT	182 bp	XM_015290060.4
*Bcl2*	Forward: CTTCTGCCATTCAAGACGAGTTCTG Reverse: GCAATACCTCTGGAGACCTGTACC	105 bp	XM_005028719.1
*β-actin*	Forward: TGATGGACTCTGGTGATGGTGTTAC Reverse: CTCTCGGCTGTGGTGGTGAAG	163 bp	NM_205518.1

*FAS*, fas cell surface death receptor; *ACC*, acetyl-CoA carboxylase; *SREBP1*, sterol regulatory element binding transcription factor 1; *CYP7A1*, cytochrome P450 family 7 subfamily A member 1; *CaM*, calmodulin; *LXRs*, nuclear receptor subfamily 1 group H member 3; *FSHR*, follicle stimulating hormone receptor; *LHR*, luteinizing hormone receptor; *TRPV1*, transient receptor potential cation channel subfamily V member 1; *PPARα*, peroxisome proliferator activated receptor α; *StAR*, steroidogenic acute regulatory protein; *CYP11A1*, cytochrome P450 family 11 subfamily A member 1; *CYP19A1*, cytochrome P450 family 19 subfamily A member 1; *Bax*, transmembrane BAX inhibitor motif containing 1; *Bcl2*, B-cell lymphoma 2.

### Statistical analysis

All data analysis on production performance, egg quality, reproductive hormones, follicle development, lipid metabolism parameters, antioxidant capacities and genes expression were performed using one-way analysis of ANOVA (SPSS version 20.0) followed by significant differences via Duncan`s analysis. The pen was as an experimental unit for production performance, whereas the individual broiler was the experimental unit for the other parameters. All values were expressed means and standard error of the mean (**SEM**) of the mean and *P* < 0.05 indicated significant differences.

## Results

### Production performance

Compared with the control group and PF group, CAP supplementation increased ADFI and decreased the ratio of feed to egg of laying hens during the experiment periods of 1-4 W, 5-8 W and 1-8 W (*P* < 0.05, Table 3). However, there were no differences in average egg weight among all experimental groups (*P* > 0.05). In addition, CAP supplementation induced increases in egg mass and egg production of laying hens in comparison with those in the control group and PF group (*P* < 0.05).

**Table 3:** Effect of capsaicin supplementation on production performance of Changshun green-shell hens at late-laying period (*n* = 10).

Items	Control	CAP	PF	SEM	*P* value
1-4 W					
ADFI (g)	116.78^b^	123.10^a^	117.56^b^	0.562	<0.001
Average egg weight (g)	59.40	59.63	59.77	0.112	0.432
Egg Mass (g/hen d)	33.73^b^	38.87^a^	34.67^b^	0.692	0.002
Egg production (%)	56.75^b^	65.19^a^	58.05^b^	1.163	0.003
Feed to egg ratio (g/g)	2.70^a^	2.40^b^	2.63^a^	0.030	<0.001
5-8 W					
ADFI (g)	103.33^b^	110.70^a^	105.44^b^	0.730	0.996
Average egg weight (g)	59.25	59.59	59.26	0.119	0.434
Egg Mass (g/hen d)	27.57^b^	36.96^a^	28.90^b^	0.833	<0.001
Egg production (%)	56.75^b^	65.19^a^	58.05^a^	1.379	0.005
Feed to egg ratio (g/g)	2.83^a^	2.40^b^	2.80^a^	0.001	0.961
1-8 W					
ADFI (g)	110.06^b^	116.90^a^	111.50^b^	0.616	<0.001
Average egg weight (g)	59.32	59.61	59.51	0.092	0.452
Egg Mass (g/hen d)	30.65^b^	37.79^a^	31.79^b^	0.689	<0.001
Egg production (%)	51.64^b^	63.40^a^	53.41^b^	1.147	0.003
Feed to egg ratio (g/g)	2.77^a^	2.40^b^	2.72^a^	0.038	<0.001

Control, basal diet; CAP, basal diet with 150 mg/kg capsaicin; PF, basal diet with the feed consumed equal to the CAP group on the previous day. CAP, capsaicin. ADFI, average daily feed intake. Data were presented with mean and SEM. SEM, standard error of the mean. Different superscript letters in the same row indicates significant differences (*P* < 0.05).

### Egg quality

The data in Table 4 showed that dietary supplemented with CAP did not affect albumen ratio, yolk ratio and egg shape index of Changshun green-shell laying hens at the 4th and 8th week of the experiment when compared with the control group and the PF group (*P* > 0.05). Moreover, no differences were observed in albumen ratio, yolk ratio and egg shape index of Changshun green-shell laying hens at 4th and 8th week of the experiment between the control group and PF group (*P* > 0.05). Nevertheless, in comparison with the control group, 150 mg/kg CAP supplementation improved egg yolk color of hens (*P* < 0.05). In addition, Haugh unit was higher in 150 mg/kg CAP group than those in the control group and PF group (*P* < 0.05). However, no differences were observed in yolk color or Haugh unit of hens between the control group and PF group (*P* > 0.05).

**Table 4:** Effect of capsaicin supplementation on egg quality of Changshun green-shell hens at late-laying period (*n* = 20).

Items	Control	CAP	PF	SEM	*P* value
Week 4					
Yolk color	12.54^b^	13.39^a^	12.37^b^	0.096	<0.001
Albumen ratio (%)	48.48	49.84	48.70	0.710	0.699
Yolk ratio (%)	32.99	32.58	33.16	0.254	0.654
Egg shape index (%)	1.30	1.29	1.29	0.008	0.858
Haugh unit	80.60^b^	86.23^a^	81.27^b^	0.921	0.021
Week 8					
Yolk color	11.27^b^	12.32^a^	11.35^b^	0.114	<0.001
Albumen ratio (%)	50.05	50.15	51.15	1.138	0.382
Yolk ratio (%)	32.50	31.55	31.85	0.644	0.834
Egg shape index (%)	1.31	1.35	1.32	0.009	0.200
Haugh unit	78.96^b^	83.37^a^	79.54^b^	0.501	0.032

Control, basal diet; CAP, basal diet with 150 mg/kg capsaicin; PF, basal diet with the feed consumed equal to the CAP group on the previous day. CAP, capsaicin. Data were presented with mean and SEM. SEM, standard error of the mean. Different superscript letters in the same row indicates significant differences (*P* < 0.05).

### Reproductive hormones in serum

Dietary supplemented with 150 mg/kg CAP increased the contents of FSH and E_2_ in serum of Changshun green-shell laying hens in comparison with the control group (*P* < 0.05, Table 5). In addition, the contents of LH and P_4_ in serum of Changshun green-shell hens at late-laying period were elevated by dietary CAP supplementation in comparison with those in the control group and PF group (*P* < 0.05). However, between the control group and PF group, no significant differences were observed in the concentrations of FSH, E_2,_ P_4_ or LH (*P* > 0.05).

**Table 5:** Effect of capsaicin supplementation on serum reproductive hormones contents of Changshun green-shell hens at late-laying period (*n* = 10).

Items	Control	CAP	PF	SEM	*P* value
FSH (mIU/mL)	87.54^b^	97.02^a^	88.51^b^	1.611	0.022
LH (mIU/mL)	4.04^b^	6.80^a^	4.20^b^	0.266	<0.001
P_4_ (ng/mL)	0.38^b^	0.64^a^	0.40^b^	0.037	0.003
E_2_ (ng/mL)	332.74^b^	423.64^a^	362.30^b^	10.508	<0.001

Control, basal diet; CAP, basal diet with 150 mg/kg capsaicin; PF, basal diet with the feed consumed equal to the CAP group on the previous day. CAP, capsaicin. FSH, follicle stimulating hormone releasing hormone; LH, luteinizing hormone; P_4_, progesterone; E_2_, Estradiol. Data were presented with mean and SEM. SEM, standard error of the mean. Different superscript letters in the same row indicates significant differences (*P* < 0.05).

### Reproductive organ and ovarian follicle development

Compared with the control group, the ovary relative weight was higher in 150 mg/kg CAP group (*P* < 0.05, Table 6). But there were no differences in oviduct relative weight among the control group, CAP group and PF group (*P* > 0.05). In similar with this, no significant differences in large yellow follicles number were observed in the control group, CAP group and PF group (*P* > 0.05). However, 150 mg/kg CAP supplementation increased small yellow follicles number, as well as large yellow follicle relative weight and small yellow follicle relative weight (*P* < 0.05).

**Table 6:** Effect of capsaicin supplementation on reproductive organ and ovarian follicle development of Changshun green-shell hens at late-laying period (*n* = 10).

Items	Control	CAP	PF	SEM	*P* value
Ovary relative weight (g/g)	18.90^b^	22.15^a^	19.81^b^	0.385	<0.001
Oviduct relative weight (g/g)	23.63	24.70	23.74	0.611	0.752
Large yellow follicles number	5.75	5.96	5.88	0.216	0.208
Small yellow follicles number	10.13^b^	12.88^a^	10.75^b^	0.435	0.018
Large yellow follicle relative weight (g/g)	79.91^b^	85.35^a^	81.26^b^	0.844	0.016
Small yellow follicle relative weight (g/g)	2.73^b^	3.19^a^	2.76^b^	0.076	0.015

Control, basal diet; CAP, basal diet with 150 mg/kg capsaicin; PF, basal diet with the feed consumed equal to the CAP group on the previous day. Data were presented with mean and SEM. SEM, standard error of the mean. Different superscript letters in the same row indicates significant differences (*P* < 0.05).

### Lipid parameters in plasma

**Fig. 1:**
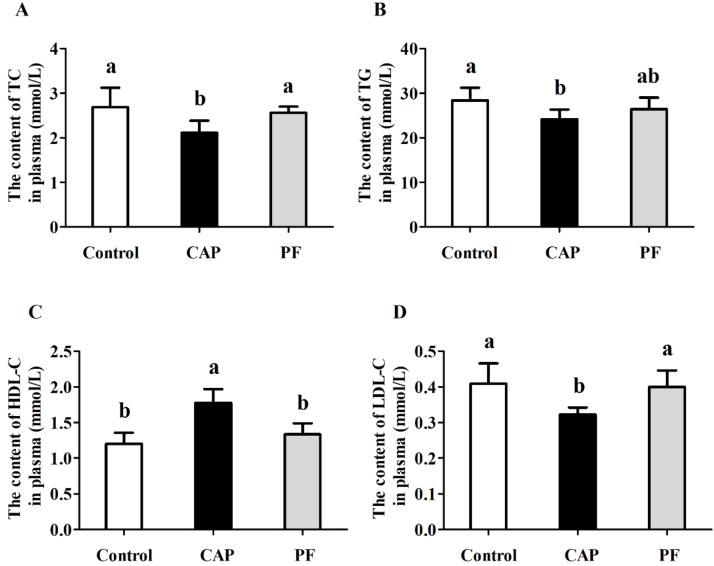
Effects of capsaicin supplementation on lipid metabolism indices in plasma of laying hens at the late laying period (*n* = 10). Control, basal diet; CAP, basal diet with 150 mg/kg capsaicin; PF, basal diet with the feed consumed equal to the CAP group on the previous day. CAP, capsaicin. TC, total cholesterol; TG, triglyceride; HDL-C, high-density lipoprotein cholesterol; LDL-C, low-density lipoprotein cholesterol. Data were presented with mean±standard deviation. Different small letter above the column indicates significant differences (*P* < 0.05).

As displayed in Fig. 1, no differences in TC, TG, HDL-C or LDL-C were observed between the control group and PF group (*P* > 0.05). In contrast, 150 mg/kg CAP led to the decreased contents of TC, TG and LDL-C in plasma of laying hens (*P* < 0.05). However, in comparison with the control group and PF group, the increased concentration of HDL-C was induced by 150 mg/kg CAP addition (*P* < 0.05).

### Antioxidant capacity

**Fig. 2:**
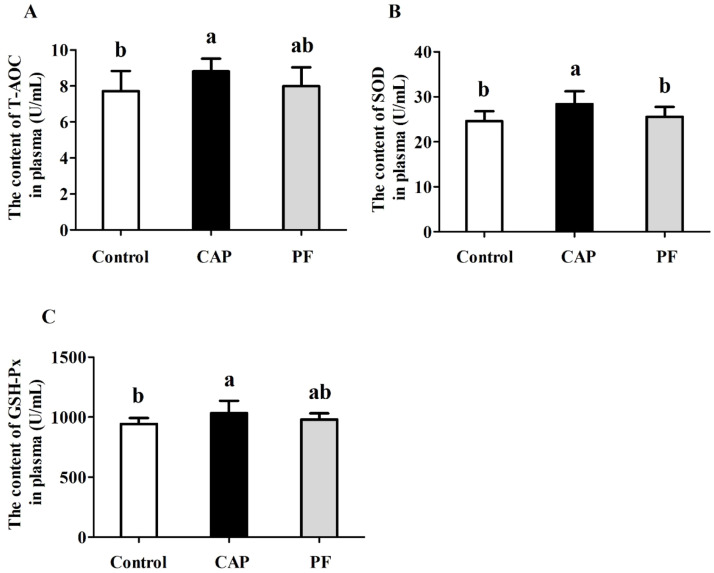
Effects of capsaicin supplementation on antioxidant capacities in plasma of laying hens at later laying period (*n* = 10). Control, basal diet; CAP, basal diet with 150 mg/kg capsaicin; PF, basal diet with the feed consumed equal to the CAP group on the previous day. CAP, capsaicin. T-AOC, total antioxidant capacity; SOD, superoxide dismutase; GSH-Px, glutathione peroxidase. Data were presented with mean±standard deviation. Different small letter above the column indicates significant differences (*P* < 0.05).

It was shown that 150 mg/kg CAP addition resulted in increases in the contents of T-AOC, SOD and GSH-Px in plasma of Changshun green-shell hens at later laying period in comparison with the control group (*P* < 0.05, Fig. 2). In contrast, no differences were found in the contents of T-AOC, SOD or GSH-Px of layers between PF group and the control group (*P* > 0.05). Besides, there were no differences in contents of T-AOC and GSH-Px in plasma between CAP group and PF group (*P* > 0.05).

### Cecal microbial structure and community

**Fig. 3:**
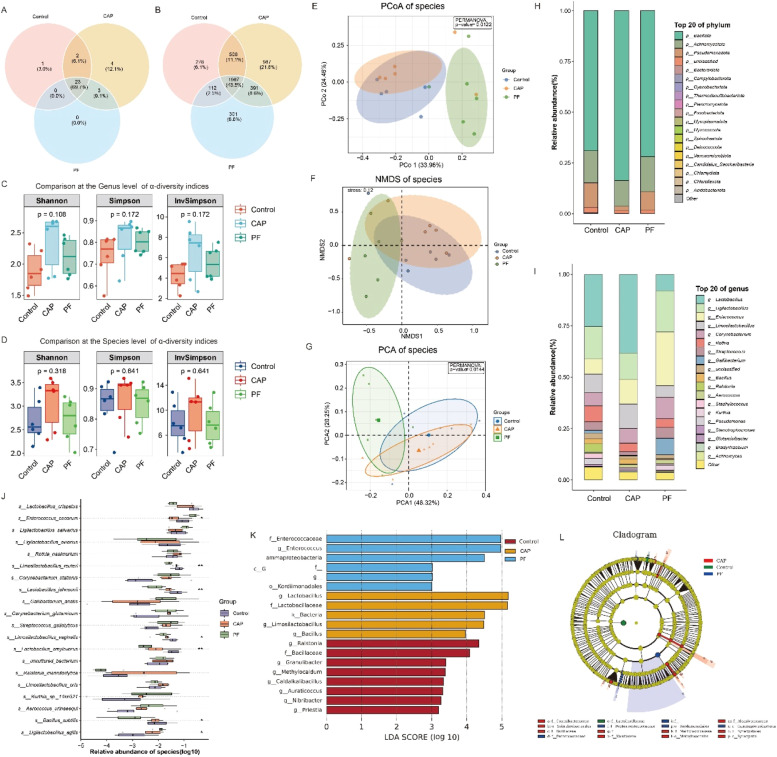
Microbial community structure in the cecal contents of layers (*n* = 6). (A-B) Venn diagram of microorganisms identified at different taxonomic levels. (C-D) Alpha diversity analysis at the genus level with Invsimpon, Simpson, and Shannon indices calculated for 18 microbial samples. The horizontal bars within the boxes represent the mean values. The top and bottom of the boxes represent the upper and lower quartiles, respectively. Double asterisks indicate *P* < 0.01**, and triple asterisks indicate *P* < 0.001***. (E, F, G) NMDS, PCoA, and PCA plots. Different colors represent samples from different groups. The distance between points on the plot indicates the similarity in microbial community composition and abundance among all samples. (H, I) Stacked plot of species abundance for the top 20 genera across different groups. (J) Analysis of species level differences between different groups. (K-L) Lefse analysis of cecal microbial variations. Control, basal diet; CAP, basal diet with 150 mg/kg capsaicin; PF, basal diet with the feed consumed equal to the CAP group on the previous day. CAP, capsaicin.

The cecal microbiota composition and community structure were assessed in the control, CAP, and PF groups (Fig. 3A-3L) . The results showed that 33 phyla, 63 classes, 135 orders, 303 families, 1,044 genera, and 4,565 species were identified in the three groups. As shown in Fig. 3A and 3B, 23 phyla and 1,987 species-level taxa were common among the three groups. The results of α-diversity analysis indicated that no significant differences in genus and species level were observed among the three groups (Fig. 3C and 3D). The analysis of β-diversity revealed that significant differences in the bacterial community structure among the three groups. PCA analysis showed that the control group, CAP group, and PF group could be distinctly separated, indicating significant differences in the cecal microbiota diversity of layers among the three treatments (PERMANOVA, *P* < 0.01). Similarly, the PCoA model also demonstrated that clear differences were found among the three groups (PERMANOVA, *P* < 0.05) (Fig. 3F and 3G). NMDS analysis yielded consistent results (stress = 0.12). At the phylum and genus levels, the analysis in the composition of cecal microbiota among the three groups showed that at the phylum level, the three main phyla observed were *Bacillota* (previously named *Firmicutes*), *Actinomycetota*, and *Puseudomonadota*. The top 10 species abundances at the genus level were as follows: g__*Lactobacillus*, g__*Ligilactobacillus*, g__*Enterococcus*, g__*Limosilactobacillus*, g__*Corynebacterium*, g__*Rothi*a, g__*Streptococcus*, g__*Gallibacterium*, g__*Bacillus*, g__*Ralstonia (*Fig. 3H and 3I). Through LDA effect size analysis, it was found that species with significant differences in abundances could be used as biomarkers in the CAP group, including f_L *lactobacillaceae*, g.L *lactobacillus*, and g.L *limosilactobacillus*. In the control group, g__*Auraticoccus*, g__*Granulibacter*, g__*Priestia*, g__*Caldalkalibacillus*, g__*Methylocaldum*, f__*Bacillaceae*, g__*Ralstonia*, g__*Nibribacter* could serve as a biomarker. In the PF group, c__*Gammaproteobacteria*, f__*Enterococcaceae*, o__*Kordiimonadales*, g__*Enterococcus* could be used as biomarkers (Fig. 3K and 3L). Subsequently, at the species level, it was showed that significant differences were found in s__*Enterococcus_cecorum*, s__*Limosilactobacillus_reuteri*, s__*Lactobacillus_john- sonii*, s__*Limosilactobacillus_vaginalis*, s__*Lactobacillus_amylovoru*, s__*Bacillus_ subtilis*, s__*Ligilactobacillus_agilis* among three treatments (Fig. 3J).

### SCFA profile of cecal digesta

**Fig. 4:**
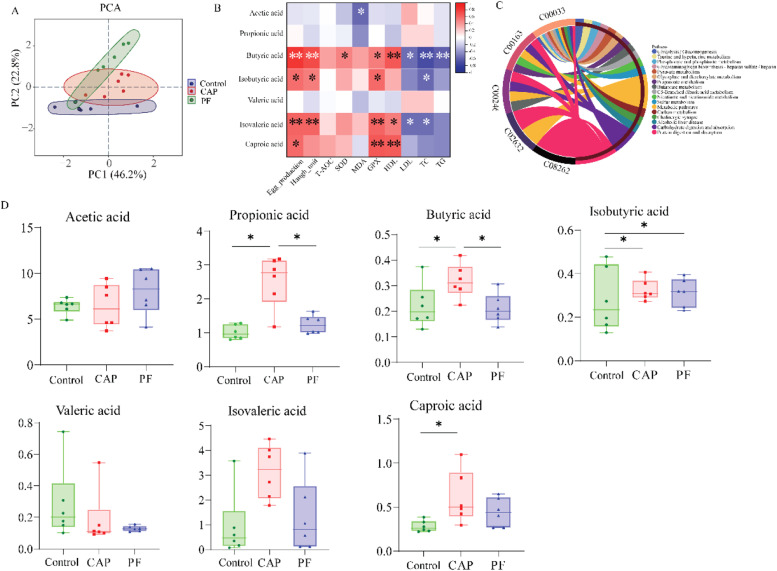
Effects of capsaicin supplementation on the contents of SCFA in cecal samples of laying hens (*n* = 6). (A) PCA analysis; (B) Spearman correlation heatmap between cecal SCFA, egg quality and serum parameters; (C) KEGG annotation chart; (D) SCFA contents. Control, basal diet; CAP, basal diet with 150 mg/kg capsaicin; PF, basal diet with the feed consumed equal to the CAP group on the previous day. CAP, capsaicin. Single asterisks indicate *P* < 0.05*.

As presented in Fig. 4, PCA analysis found that there were significant differences among the control, CAP and PF group (Fig. 4A). The results of correlation analysis showed that significant positive relationships were found between metabolites. KEGG annotation of metabolites revealed that protein digestion and absorption (ko04974) contained five metabolites; metabolic pathways (ko01100) contained four metabolites; carbohydrate digestion and absorption (ko04973) contained three metabolites (Fig. 4C). The concentrations of propionic acid and butyric acid were higher in CAP group than that in the control group and PF group (*P* < 0.05). Besides, compared with the control group, the content of isobutyric acid in CAP and PF group were elevated (*P* < 0.05). In addition, CAP treatment induced a higher content of caproic acid compared with the control group (*P* < 0.05). It was observed that butyric acid, isobutyric acid and isovaleric acid were significantly positively correlated with egg production and Haugh unit (*P* < 0.05, Fig. 4B). Moreover, butyric acid, isovaleric acid and caproic acid were positively associated with GPx and HDL-C (*P* < 0.05). Additionally, caproic acid and isobutyric acid were significantly positive correlated with egg production and GPx, respectively (*P* < 0.05). However, butyric acid was negatively associated with the concentrations of LDL-C, TC and TG (*P* < 0.05). Isovaleric acid was also negatively associated with the concentrations of LDL-C and TC (*P* < 0.05). In addition, a negative association was found between isobutyric acid and the TC content (*P* < 0.05).

### Liver transcriptomic analysis of laying hens

**Fig. 5:**
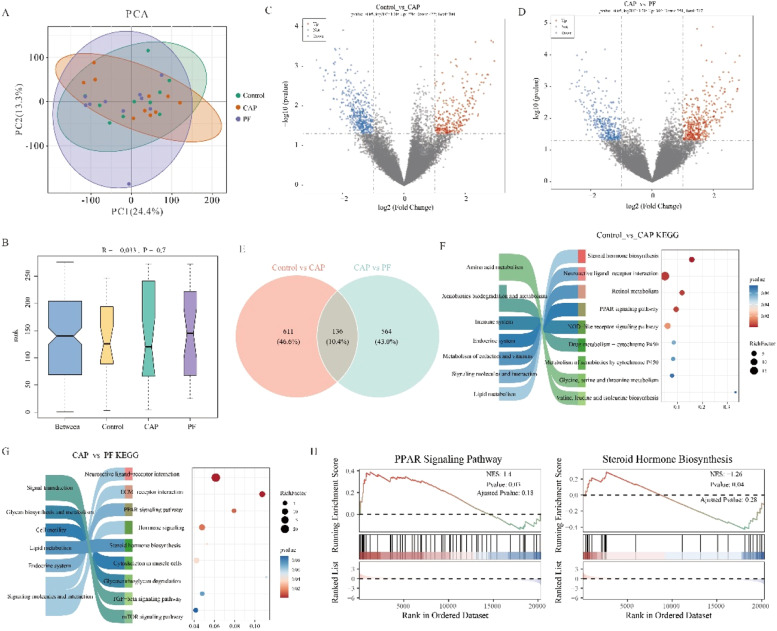
Transcriptome analysis of laying hens (*n* = 10). mRNA PCA analysis and correlation heatmap (A-B); Differential mRNA volcano plot (C-D); Differential mRNA volcano expression pattern and enrichment analysis (E); KEGG analysis (F-G); the results of GSEA enrichment analysis between the control group and CAP group (H). Control, basal diet; CAP, basal diet with 150 mg/kg capsaicin; PF, basal diet with the feed consumed equal to the CAP group on the previous day. CAP, capsaicin.

The results of PCA analysis revealed that there were significant differences among three groups. Combined with the correlation plot between the samples, it was found that there was a high degree of intra-group reproducibility. The different groups were clearly separated on PCA, indicating a high difference between the groups (Figs. 5A and 5B).

To explore the mRNA differences between the two groups, DEseq2 software was used to analyze and screen the differential mRNAs between Control_vs_CAP and Control_vs_PF groups according to the threshold value of *P* < 0.05 & |logFC|>1. The results showed that transcriptome analysis revealed 700 differentially expressed mRNAs in the CAP-treated group in comparison with the control group, with 278 differential mRNAs up-regulated and 422 differential mRNAs down-regulated as (Fig. 5C). Comparative analysis of the PF group relative to the control group yielded 747 significant mRNAs expression, of which 396 differential mRNAs were up-regulated and 351 differential mRNAs were down-regulated (Fig. 5D). The two combinations of differential genes were done as venn plots as in Fig. 5E, and the results found that 136 genes were shared differential genes. The results of KEGG showed that enrichment analysis of differential metabolites between the control group and CAP group mainly focused on steroid hormone biosynthesis, Neuroactive ligand-receptor interaction, Retinol metabolism and PPAR signaling pathway (Fig. 5F). Moreover, the results of KEGG showed that enrichment analysis of differential metabolites between the control group and CAP group were Neuroactive ligand-receptor interaction, ECM-receptor interaction, PPAR signaling pathway and Hormone signaling (Fig. 5G). In addition, CAP supplementation positively regulated PPARα signaling pathway but negatively influenced steroid hormone biosynthesis.

### mRNA expression related to lipid metabolism

**Fig. 6:**
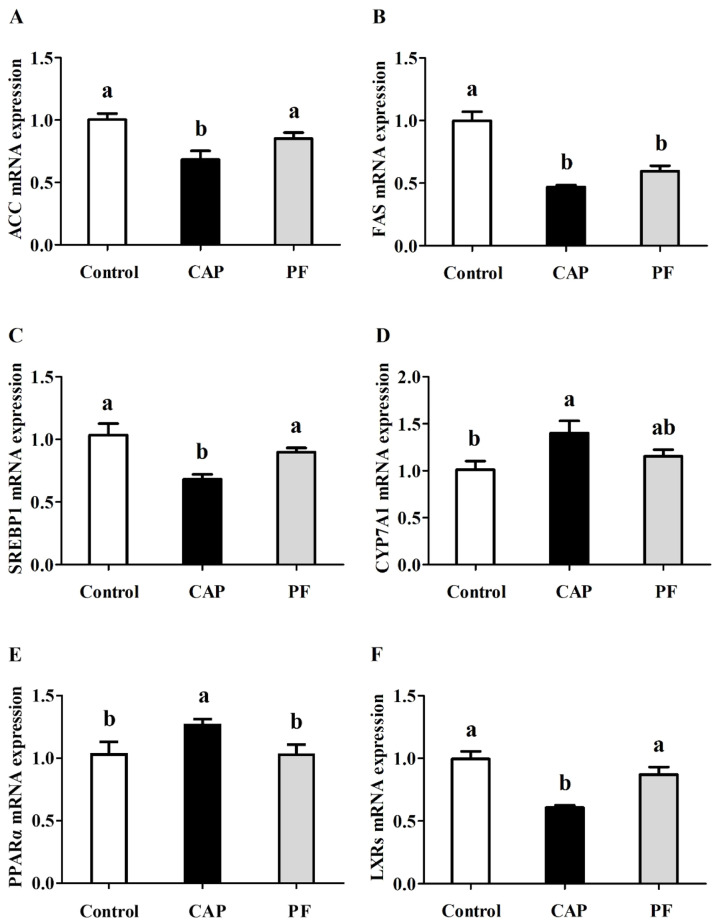
Effects of capsaicin supplementation on mRNA expressions of *ACC* (A), *FAS* (B), *SREBP1* (C), *CYP7A1* (D), *PPARα* (E) and *LXRs* (F) of laying hens at later laying period (*n* = 10). Control, basal diet; CAP, basal diet with 150 mg/kg capsaicin; PF, basal diet with the feed consumed equal to the CAP group on the previous day. CAP, capsaicin. *ACC*, acetyl-CoA carboxylase; *FAS*, fatty-acid synthase; *SREBP1*, sterol regulatory element binding protein-1; *CYP7A1*, cholesterol 7a-hydroxylase; *PPARα*, peroxisome proliferator-activated receptor α; *LXRs*, nuclear receptor subfamily 1 group H member 3. Data were presented with mean±standard deviation. Different small letter above the column indicates significant differences (*P* < 0.05).

As shown in Fig. 6, mRNA expressions of *ACC, FAS, SREBP-1* and *LXRs* of layers were downregulated by CAP administration (*P* < 0.05). However, mRNA expressions of *PPARα* and *CYP7A1* in liver of layers in CAP group were enhanced compared with the control group (*P* < 0.05). Besides, there were no *CYP7A1* and *PPARα FAS* mRNA expressions between the CAP group and the PF group (*P* > 0.05).

### mRNA expression related to follicle development

**Fig. 7:**
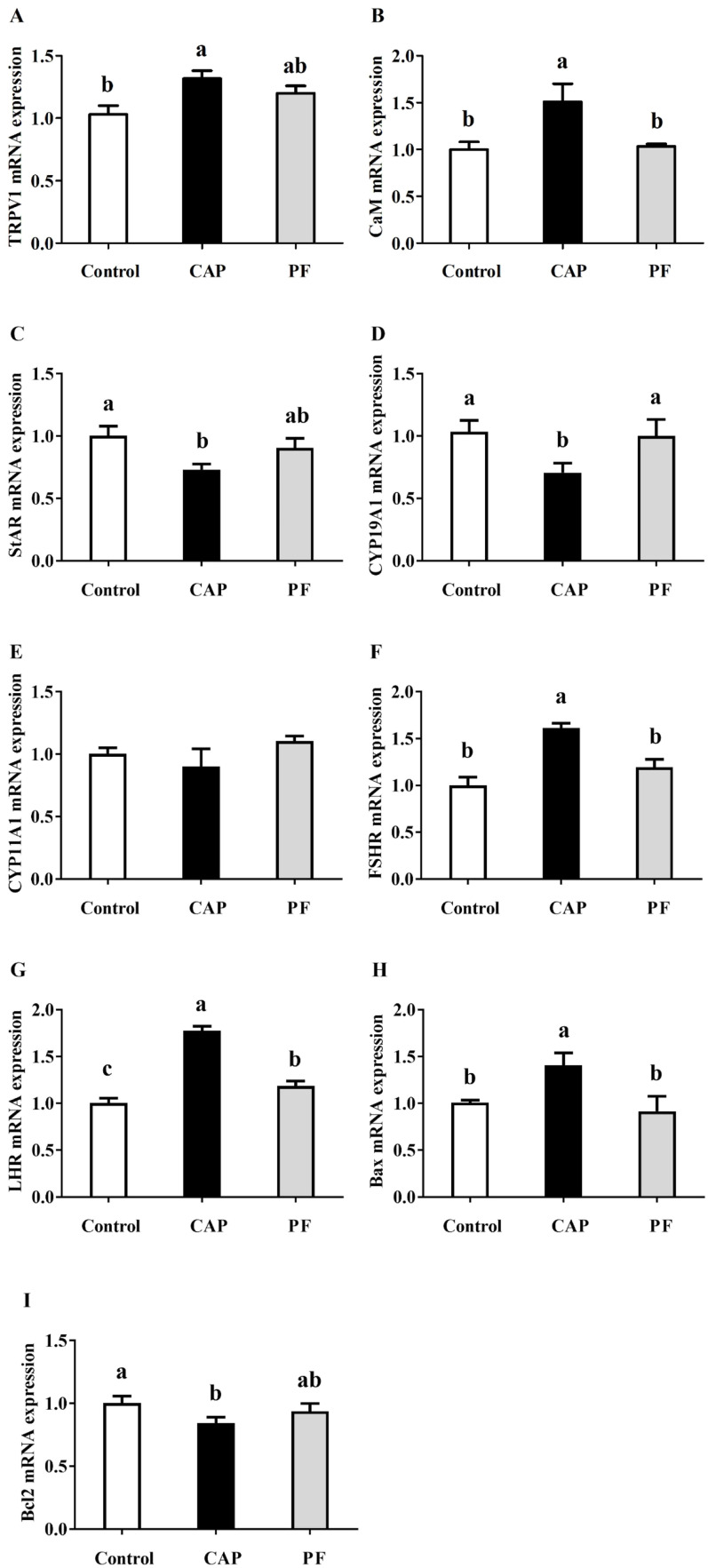
Effects of capsaicin supplementation on ovarian mRNA expressions of genes related to follicle development of laying hens at later laying period (*n* = 10). Control, layers were provided with the basal diet containing no capsaicin; Control, basal diet; CAP, basal diet with 150 mg/kg capsaicin; PF, basal diet with the feed consumed equal to the CAP group on the previous day. CAP, capsaicin. *TRPV1*, transient receptor potential vanilloid 1; *CaM*, Calmodulin; *StAR*, steroidogenic acute regulatory protein; *CYP19A1*, cytochrome P450 family 19 subfamily A member 1; *CYP11A1*, cytochrome P450 family 11 subfamily A member 1; *FSHR*, Follicle-stimulating hormone receptor; *LHR*, Luteinizing hormone receptor; *Bax*, transmembrane BAX inhibitor motif containing 1; *Bcl2*, B-cell lymphoma 2 protein. Data were presented with mean±standard deviation. Different small letter above the column indicates significant differences (*P* < 0.05).

The results showed that the layers at the late-period cycle in the CAP group exhibited higher mRNA expressions of *TRPV1, CaM, FSHR* and *LHR* in ovary than those in the control group (*P* < 0.05, Fig. 7). Moreover, compared with the control group, the decreased mRNA expressions of *CYP19A1* were observed in layers in the CAP group (*P* < 0.05). But there was no difference in *CYP11A1* mRNA expression among three groups (*P* > 0.05). In addition, CAP treatment elevated mRNA expression of *Bax*, but decreased *Bcl-2* mRNA expression (*P* < 0.05).

## Discussion

Egg production is one of critical biological and economic traits, which directly influences the productivity and profitability of commercial layer industry (Zhao et al., 2023). However, late-phase laying hens are generally susceptible to various stresses owing to the weakened function of reproductive system, imbalance of redox process and the disturbance of lipid metabolism, which results in the decline in both productive performance and egg quality (Xu et al., 2023; Zhang et al., 2023). In our study, it was found that 150 mg/kg CAP supplementation led to an increase in ADFI and a decrease in the ratio of feed to egg of hens at 1-4 W, 5-8 W and 1-8 W when compared with those in the control group. In addition, in comparison with the control group, egg mass and egg production were significantly enhanced by CAP addition. However, no differences in the indexes aforementioned between the control group and PF group were observed. According to existing evidence, the study about CAP supplementation on production performance of hens was scarce. The results in a previous study showed that 0.5 % red pepper powder supplementation in diets of hens (32-40 weeks of age) ameliorated production performance, evidenced by the elevated egg production, the increased egg mass as well as the decreased feed conversion ratio (Abou-Elkhair et al., 2018). Similarly, Saleh et al. (2021) also suggested that, in comparison with the control group, 0.4 % paprika supplementation significantly increased egg production and decreased the feed conversion ratio. In addition, it was demonstrated that ducks receiving diets containing 150 mg/kg CAP exhibited the increased feed intake and a trend to the higher egg weight and egg mass (Liu et al., 2021a). Taken together, the information mentioned above indicated that CAP supplementation contributed to improving production performance of laying hens at the late period of laying cycle.

In commercial layer production, maintaining high egg quality is essential for profitability and brand reputation. The Haugh unit, calculated from the ratio of the height of albumen to egg weight, is considered as a key indicator of albumen quality (Abou-Elkhair et al., 2018). Moreover, yolk color is one of the main parameters by which the quality of eggs is judged (Beardsworth and Hernandez, 2004). Abou-Elkhair et al. (2018) demonstrated that dietary supplementation with 0.5 % red pepper powder significantly improved the Haugh unit of eggs in laying hens. Similarly, Sözcü (2019) reported that 1.0 % red pepper powder supplementation significantly enhanced egg Haugh units of hens. In our present study, it was found that inclusion of 150 mg/kg CAP in laying hen diet had the highest value of Haugh unit among three treatments. It was suggested that the bioactive ingredients derived from herbal plants exhibited benefits of maintaining magnum and uterus, and promoting the albumen secretion in laying birds (Radwan et al., 2008), indicating that CAP could protect the magnum of hens contributing to improving albumen quality. Similarly, the dietary inclusion CAP darkened yolk color compared with the control group. In agreement with the results of ours, available accumulated evidences revealed that yolk color of eggs from hens receiving diet containing natural chili powder was enhanced (Lokaewmanee et al., 2013; Rossi et al., 2015). In addition, carotenoids in chili peppers, functioning as natural colorants, could enhance yolk pigmentation due to their high bioavailability and yolk deposition efficiency (Chen et al., 2015). Taken together, the information aforementioned indicated that CAP inclusion in diet could improve albumen quality and yolk color of hens at the late phase of laying cycle.

The development of ovaries is the key element regulating egg production activities of hens (Chen et al., 2021b). Our results showed that CAP supplementation increased the ovary relative weight, implying that CAP contributed to promoting the development of ovary. It has been demonstrated that CAP could stimulate follicle development (Zık et al., 2010). Besides, the follicular development of hens was stimulated by diets containing red hot pepper (Özer et al., 2005). The results in our present study suggested that CAP supplementation increased the large yellow follicle relative weight and small yellow follicle relative weight combined with the small yellow follicles numbers. Liu et al. (2021a) also reported that 150 mg/kg CAP supplementation in diets of the aged laying duck increased the ratio of large yellow follicle weight to ovarian weight and the ratio of small yellow follicle weight to ovarian weight. The follicle development is regulated by hormones secreted by the “hypothalamus-pituitary-gonadal” axis (Zhao et al., 2023). It is well-known that gonadotropins and steroid including FSH, LH, P4 and E2 are associated with the course of follicular growth and maturation, which can influence the production performance of laying hens (Niu et al., 2023; Ding et al., 2024). Accordingly, the contents of serum reproductive hormones such as FSH, LH, P4 and E2 of hens fed with diet containing 150 mg/kg CAP were higher than those in the control diet. Taken together, the information aforementioned indicated that dietary CAP supplementation could enhance the secretion of reproductive hormones in laying hens, furtherly promoting follicular development and subsequently increasing egg production rate.

It is well documented that the disruption of redox homeostasis and oxidative stress would inevitably occur leading to the excess production of reactive oxygen species (**ROS**), which ultimately resulted in lipid, protein and DNA damage (Oke et al., 2024). It has been demonstrated that anti-oxidant enzymes including SOD, CAT and GSH-Px are crucial for defending against oxidative stress-induced cellular damage (Escorcia et al., 2020). In previous studies, it was suggested that CAP exhibited a strong antioxidant effect *in vitro* and *in vivo* (Liu et al., 2015; Zhang et al., 2017). It was also found that antioxidant capacities of broilers were enhanced by natural capsicum extract supplementation, exhibited as the increased contents of T-AOC, GSH-Px and SOD in plasma. In consistent with this, our results revealed that dietary inclusion with 150 mg/kg CAP elevated the concentrations of T-AOC, SOD and GSH-Px in plasma in comparison with those in the control group, suggesting that CAP could improve antioxidant capacity of laying hens.

The liver is a vital organ playing crucial effects in regulating lipid metabolism in the egg-laying process (Chu et al., 2025). The lipid levels in blood are closely related to lipid synthesis and transport in the liver. It is important to note that TG and TC levels correlate with dietary lipid absorption, whereas HDL-C, LDL-C, and VLDL dynamics reflect endogenous lipid catabolism and circulatory redistribution (Cui et al., 2022). It was observed in our study that hens in the CAP group exhibited lower concentrations of TC, TG and LDL-C as well as higher HDL-C in plasma than those in the control group. In similar with our current results, Pi et al. (2017) demonstrated that the serum content of TC, TG and LDL-C were markedly reduced, whereas the serum HDL-C level showed a pronounced increase following CAP administration. Moreover, in ovariectomized rats, capsaicinoid supplementation (15 mg/kg/day) led to a significant decrease in the concentrations of TC, TG, and LDL in plasma (Zhang, 2013). Current evidence indicates that hepatic lipid overaccumulation in late-lay hens induces fatty liver syndrome, compromising hepatic function and yolk precursor synthesis, ultimately diminishing egg production rates (Huang et al., 2022). These findings indicated that CAP could be efficacy in suppressing liver fat accumulation and decreasing fatty liver syndrome in late-phase laying hens.

The gut microbiota, comprising diverse bacterial, archaeal, viral, and eukaryotic communities, serves as a key regulator in modulating numerous physiological processes in humans and animals (Ruesga-Gutiérrez et al., 2022). The gut microflora composition in laying hens can be influenced by breed, age, environment, and diet, with diet being the most significant factor (Liu et al., 2023). The cecal microflora in laying hens is complex and dynamic, comprising *Bacillota, Bacteroidetes, Actinobacteria, Proteobacteria*, and other bacterial phyla (Rychlik, 2020). In our present study, no significant changes in cecal microbiota alpha diversity were observed in late-laying hens following CAP administration, suggesting a dynamically balanced microbial community. However, it was found that the domain phyla were *Bacillota, Actinobacteria* and *Bacteroidota* among the three groups. In consistent with this, Song et al. (2017) demonstrated that, at the genus level, CAP administration altered the abundances of *Bacteroides*. At the species level, CAP supplementation altered the relative abundances of *Lactobacillus, Limosilactobacillus* and *Ligilactobacillus* communities in cecum, which was demonstrated to be associated with production performance of hens at the late-phase laying period (Liu et al., 2023). In addition, it was also found that 0.6 % *Lactobacillus plantarum* supplementation increased laying rate of laying hens (Choe et al., 2012). The information mentioned above indicated that CAP addition altered cecal microbiota composition, furtherly contributing to improving the production performance of laying hens. Moreover, it has been demonstrated that the *Bacteroides* phylum produces acetate and propionic acid as the primary metabolic products (Huang et al., 2023), whereas the main productions of *Lactobacillus* are acetic acid, propionic acid and butyric acid (Thananimit et al., 2022). In accordance with this, the results of our present study revealed that CAP administration enhanced the concentrations of propionic acid, butyric acid and isobutyric acid compared with the control group. In a previous study, it has been demonstrated that SCFAs could function as key gut-live axis mediators (Yin et al., 2023). Consequently, in a previous study, it was suggested that propionate could be acted as a precursor for hepatic gluconeogenesis thereby decreasing the hepatic synthesis of cholesterol (Chakraborti, 2015), which was in consistent with the decreased TG content in serum induced by CAP supplementation. Besides, propionate was proven to reduce cholesterol synthesis in the live and improve lipid metabolism (Markowiak-Kopeć and Śliżewska, 2020), and butyric acid was found to be negatively correlated with lipogenesis in the liver (Chen et al., 2023). Indeed, the result of correlation analysis suggested that there were negative effects between the contents of butyric acid and the concentrations of LDL-C, TC and TG in plasma serum. Interestingly, in our present study, CAP supplementation elevated the content of the butyric acid, but reduced contents of TC, TG, and LDL-C in serum of layers. As previously stated, the inclusion of CAP could positively alter the composition of gut microbiota, enhance the concentrations of SCFA, and thereby modulate lipid metabolism of layers at the late-laying period.

ACC and FAS, the two key rate-limiting enzymes modulating de novo lipogenesis, are transcriptionally regulated by LXRs, PPARα, and SREBP-1 (Song et al., 2019). Furthermore, PPARα and SREBP-1 transcriptionally active de novo free fatty acid synthesis by inducing the expression of rate-limiting enzymes particularly FAS and ACC (Ha et al., 2016). LXRα, acting as a ligand-activated nuclear receptor, regulates SREBP-1 to stimulate ACC and FAS expression, while PPARα conversely regulates fatty acid β-oxidation (Wang et al., 2020). In accordance with this, our results demonstrated that CAP induced the downregulation of mRNA expressions of *ACC, FAS, SREBP-1* and *LXRs* in liver of layers. However, CAP supplementation upregulated mRNA expression of *PPARα* in liver. Meanwhile, the upregulated mRNA expression of *CYP7A1* in liver was induced by CAP administration. CYP7A1, responsible for the rate-limiting step in the classic pathway of bile acid synthesis, participates in lipid metabolism of liver (Feng et al., 2024). In a previous study, it was found that rats administrated with CAP (15 mg/kg body weight) decreased mRNA expression of *CYP7A1* in liver. CAP could exhibit positive effects on lipid metabolism and prevent obesity through regulating the expressions of *PPARα, SREBP-1* and *FAS* (Zhang et al., 2013). Xiao et al. (2025) found that layers receiving diets supplemented with 9 % chili meal exhibited lowered mRNA expressions in *ACC, SREBP-1* and *FAS* in comparison with those fed with low protein diet. In contrast, the elevated mRNA expression of *PPARα* was induced by 9 % chili meal supplementation. Collectively, these findings indicated that CAP could contribute to attenuating hepatic lipid accumulation of laying hens by suppressing lipogenesis and upregulating the expression of gene involved in fatty acid degradation.

Previous studies established that TRPV channel, a nonspecific Ca²⁺-permeable ion channel modulated by phosphoinositide, could mediate behavioral in response to diverse external stimuli including chemicals, mechanical forces, and temperature changes (Yang et al., 2010). The available evidence revealed that Ca^2+^ regulated cAMP synthesis (Halls and Cooper, 2011). Interestingly, CAP acted as a highly selective TRPV1 agonist, whose activation depends on Ca^2+^/calmodulin-mediated regulation (Liu et al., 2021a). Moreover, the Ca^2+^ mediated signaling pathway are involved in follicle selection and maturation of laying birds (Chen et al., 2020; Liu et al., 2021a). It has been demonstrated that CAP could induce the activation of TRPV1 (Zhang et al., 2013). Liu et al. (2021a) reported that CAP addition in laying duck resulted in the increased mRNA expression of *CaM* in ovary. In accordance with this, the results of our study revealed that CAP supplementation elevated mRNA expression of *TRPV1* and *CaM*, but decreased *StAR* and *CYP19A1* mRNA, indicating that CAP could regulate steroid synthesis. StAR which is essential for producing steroid hormones, directly and indirectly regulates reproductive processes (Lu et al., 2024). The upregulation of StAR and CYP19A1 gene expression occurs during follicular development (Guo et al., 2019). FSH and LH act through their respective receptors FSHR and LHR, which are expressed on follicular granulosa cells in the ovaries of poultry (Sun et al., 2023). It was suggested that 1 % red hot pepper supplementation in diet increased the activity of FSH and LH cells in pituitary glands (Erdost et al., 2006). CAP treatment for 14 days downregulated the mRNA expression of *Bcl2* (Moradi et al., 2023). Our results demonstrated that CAP administration also enhanced *FSHR, LHR, Bcl-2* and *Bax* mRNA expression, suggesting that CAP could contribute to promoting follicle selection and maturation of laying hens, furtherly improving production performance.

## Conclusion

CAP supplementation in the diet of hens at the late-period laying cycle enhanced egg production rate, ameliorated yolk color and Haugh unit of eggs. Furthermore, CAP supplementation enhanced follicle growth and maturation associated with TRPV1 and Ca^2+^ signaling pathway in ovary. In addition, CAP could contribute to improving anti-oxidant capacity, cecal microbiome composition and lipid metabolism of aged-laying hens. Therefore, this study highlights the application of CAP as a functional feed additive to improve productivity in laying hens at the late-period of laying cycle.

## CRediT authorship contribution statement

**Bolin Zhang:** Conceptualization, Data curation, Funding acquisition, Investigation, Writing – original draft, Writing – review & editing. **Ning Liu:** Data curation, Investigation, Methodology, Writing – review & editing. **Xueping Shi:** Methodology. **Caichao Zhang:** Investigation, Methodology. **Yingchun Liu:** Project administration. **Di Yang:** Investigation, Methodology. **Zongzheng Liu:** Project administration. **Wei Wang:** Project administration, Resources. **Shubai Wang:** Writing – review & editing.

## Disclosures

The authors declare that they have no known competing financial interests or personal relationships that could have appeared to influence the work reported in this paper.
